# Supplementary material for: A high-resolution mRNA expression time course of embryonic development in zebrafish
Source: eLife. 2017 Nov 16;6:e30860. doi: 10.7554/eLife.30860 (PMC5690287; doi:10.7554/eLife.30860)
Supplement: Supplementary file 6. [file elife-30860-supp6.zip › biolayout-clusters-files/Cluster230-genes.html]

Cluster230


# Cluster230: Genes

| Ensembl ID | Gene Name | Chr | Start | End | Biotype |
| --- | --- | --- | --- | --- | --- |
| ENSDARG00000098059 | fkbp3 | 20 | 54464073 | 54474617 | protein\_coding |
| ENSDARG00000022569 | nkx6.1 | 21 | 19034285 | 19037195 | protein\_coding |
| ENSDARG00000025017 | prdm8 | 13 | 31162250 | 31165908 | protein\_coding |
| ENSDARG00000078155 | si:ch211-163l21.4 | 8 | 23732009 | 23754405 | protein\_coding |
| ENSDARG00000056483 | ssbp4 | 22 | 20929477 | 21021865 | protein\_coding |
| ENSDARG00000042835 | tfdp2 | 15 | 4430650 | 4537240 | protein\_coding |
